# Comparison of the dried properties of *Ganoderma lucidum* produced by the convective dryer and infrared dryer

**DOI:** 10.1038/s41598-023-39883-z

**Published:** 2023-08-03

**Authors:** Maryam Naseri, Kamyar Movagharnejad, Sara Nanvakenari

**Affiliations:** Faculty of Chemical Engineering, Babol Noshirvani University of Technology, Babol, Mazandaran Iran

**Keywords:** Diseases, Medical research, Chemistry, Engineering, Mathematics and computing

## Abstract

*Ganoderma lucidum* is a promising medicine with a high amount of antioxidants and calcium. The selection of appropriate drying process methods in food science has a chief role to reach the best final characteristics. This study aimed to investigate the effects of air velocity and temperature in the convective dryer, sample distance, and infrared power in infrared dryers on the drying kinetics and quality of *Ganoderma lucidum* slices. In addition, Response Surface Methodology based on central composition design was used to optimize and analyze drying conditions. The ranges of temperature and air velocity were 40–60 °C and 0.5–1.5 m/s, respectively in the convective drying process while the range of distance and infrared power was 4–16 cm and 500–1500 W, respectively in the infrared drying process. It is worth mentioning that antioxidant and calcium contents were greatly enhanced during the drying procedures. Moreover, the values of the total color difference ranged between 8.21 and 19.66 for the convective dryer and 8.14 and 28.85 for the infrared dryer. A kinetic study indicated that dried samples by the infrared dryer could rapidly reach equilibrium moisture content due to exposure to IR radiation. Consequently, the results indicated that the infrared dryer has better performance than the convective dryer regarding drying time, energy consumption, and amount of calcium and antioxidant.

## Introduction

The *Ganoderma lucidum* is one of the most prestigious traditional Chinese medicines and also it is one of the most prestigious ingredients of the genus oriental macrofungi polypore known as Lingzhi^1–4^. This product is used simultaneously in both medications and foodstuffs^5,6^. Several phenolic compounds in *Ganoderma lucidum* have been studied, namely gallic acid, pyrogallol, hydroxybenzoic acid, coumaric acid, cinnamic acid, protocatechuic acid, catechin, naringin, myricetin, quercetin, kaempferol, hesperetin, and formononetin^7–9^. Recently, researchers have reported that the polysaccharides are one of the most promising active components of *Ganoderma lucidum* and they apply for many purposes including anti-tumor^10–12^, antioxidant^13–16^, hypoglycemic, and immune-stimulating effects^17–22^ because of their biological activities. In the food industry and medicine, natural polysaccharides have been used for a long time. Numerous studies have been carried out about bioactive polysaccharides especially, their structures and mechanisms in diseases^23–25^. Several years of research have proved that *Ganoderma lucidum* is an immunostimulant as well as a powerful antioxidant. It is now being used as a supplement to avoid the side effects of chemotherapy and to treat cancer^25^. GLP polysaccharides exhibit a variety of biological effects, including immunomodulatory, antineurodegenerative, antidiabetic, anti-inflammatory, anticancer, and antibacterial properties. -d-glucans, in particular, are well known for their biological and physiological activity^26^. Moreover, natural polysaccharides with different curative effects have been mostly examined and even applied in therapies^27,28^. Several factors, including chemical components, molecular weight, structure, conformation, and even drying techniques, maybe affect polysaccharide antioxidant activities, especially concerning the components removed or isolated from the raw material^15,29–32^. Drying *Ganoderma* is typically an approach for longer shelf life without applying chemical preservatives and concentrating the medicinal value in the fruiting body^15,33,34^. According to literature surveys, tissue type and its position in the cell are important factors in different drying methods to figure out their effects on phenolic compounds^35^. Using and selecting the best post-harvest techniques play an important role to improve the shelf life and preserve the mushroom quality^36^. Hayati et al. found that the form of drying materials has a great impact on the retention of the water-soluble polysaccharides content and antioxidant activities of *Ganoderma lucidum*. In addition, the fruiting bodies were thus more effective in keeping thermolabile pharmaceutical active ingredients of Ganoderma with every form of drying (drying under direct sunlight, drying under the sun, covered with black fabric, oven drying, and air circulation oven drying). Moreover, it was found that the air circulation during oven drying of *Ganoderma lucidum* had the highest retention of water-soluble polysaccharides content and antioxidant activities among other drying techniques^37^. Chen et al. used a combination drying technique comprising microwave-vacuum and conventional vacuum drying methods to extract the polysaccharides and compare the results with the freeze-drying method. They indicated that the extraction quality using this combinational technique was close to that of the freeze-dried extraction quality and it was much better than that of the conventional vacuum-dried approach^34^. The convective hot air drying approach for *Ganoderma tsugae* Murrill was investigated by Chin et al. at different conditions of drying temperature, size, and airflow. They found that the Ganoderma dried at 50 °C with an air velocity of 1.401 m/s has the maximum retention of crude ganoderic acid content^33^. Afzal et al*.* investigated energy consumption and properties of dried barley by combined FIR-convection dryer^38^. They found that infrared could enhance the drying rate and decrease energy consumption during the drying process. Moreover, Taghinezhad et al. studied the drying process of Turnip slices by the convective-infrared dryer and used a smart modeling technique to optimize and model the proposed dying process^39^.

The purpose of this research is to investigate the effects of various drying techniques on the antioxidant activity of *Ganoderma lucidum*. Response Surface Methodology (RSM) is applied as a valuable statistical approach to study the impacts of different input variables which can be significant roles in the quality of the products^40–43^. The effects of different variables including air temperature, distance, infrared power, and air velocity on the process variables including drying time, energy consumption, antioxidants, calcium content, and total color difference of *Ganoderma lucidum* are investigated and optimized by RSM with central composition design (CCD). Studying the medical aspects of *Ganoderma lucidum* as a great herbal medicine such as antioxidant and calcium amounts dried by infrared and convective dryers is the novelty of the current contribution. Moreover, this research can help researchers who deal with herbal medicines how can dry materials in the best conditions.

## Materials and methods

Fresh fruiting bodies of *Ganoderma lucidum* were hand-harvested from a farm located in *Bandar-e Anzali*, a city of Gilan Province in Iran, and then transported to the laboratory, and were cut into 2 × 2 cm square slices with 2 mm thickness. The initial moisture content of the samples on a wet basis was measured to be 78.46 ± 2% W.B using the oven at 105 °C for 24 h.

### Drying methods

#### Convective dryer

**Figure 1:**
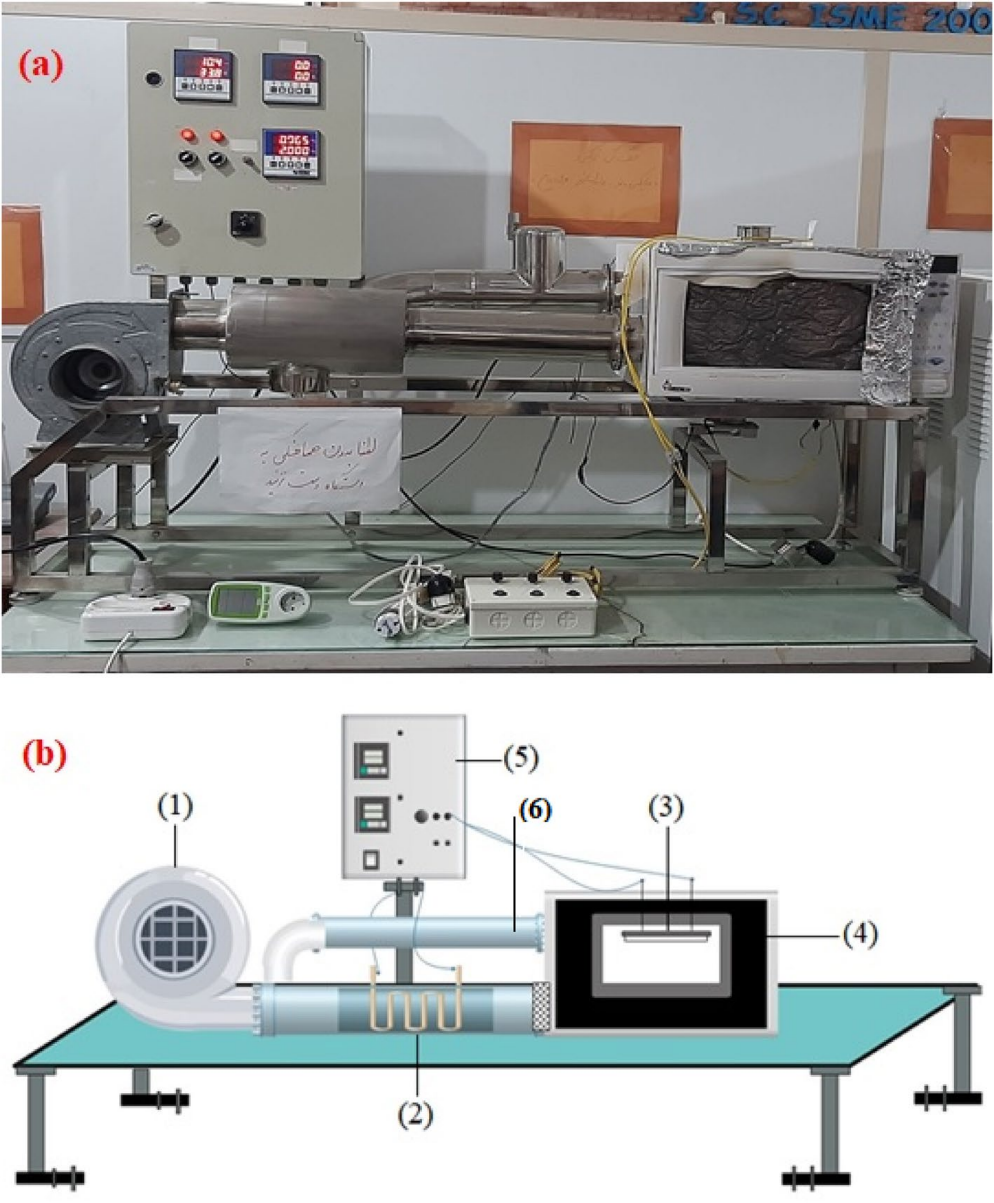
Actual (**a**) and schematic (**b**) of convective and infrared dryers: (1) blower; (2) electrical heater; (3) infrared lamps; (4) drying chamber; (5) control unit; (6) air outlet.

The experimental setup and schematic of the convective and infrared dryers used in this study are shown in Fig. 1. This setup can be used as a convective dryer when just the heater and fan are in service. Sample slices were exposed to hot air at temperature ranges between 40 and 60 °C and air velocity ranges between 0.5 and 1.5 m/s in the convective dryer. Therefore, 10 g of the raw material was measured with an electronic balance (A&D, EK-6000i, Japan), and the convective dryer was started for about 30 min to reach a steady condition before putting the samples into the convective dryer. Afterward, the weight loss of the slices was measured by an electronic balance once every 15 min until reaching the equilibrium moisture content (EMC). Moreover, it is worth mentioning that energy consumption was measured once every 15 min until reaching the EMC by power monitor (Energy model EMS-EU, England). After reaching EMC, samples were ground into powder using a mechanical grinder (Grindmatic model NO.QYX-501, Hong Kong) to use in characteristic tests.

#### Infrared dryer

As it can be seen in Fig. 1, the infrared dryer can be applied when the heater and fan are not in service and the infrared lamp should be in the service. The infrared lamp (Victory, England) with a power of 1500 W was used to supply infrared energy. The distances between the infrared lamp and the sample can be changed via wire in this system. In IR drying, the impact of different radiation intensity levels (i.e., 500, 1000, 1500 W) and lamp distance to the sample (4, 10, 16 cm) were investigated. The convective or infrared drying processes were continued until the sample's weight decreased to moisture levels less than 12 ± 2% (wet base). In more detail, the weight loss of the slices and energy consumption were measured by the electronic balance and power monitor, respectively once every 2 min until reaching the EMC. After reaching EMC, samples were ground into powder using a mechanical grinder to use in characteristic tests.

### Determination of antioxidant activity

Determination of the free radical scavenging was carried out by 2,2-diphenyl-1-picrylhydrazyl which is commonly called DPPH. According to guidelines, Miliauskas changed the Brand-Williams procedure for determining the DPPH radical scavenging potential of each extract^44,45^. The maximum absorption band of DPPH radical is 515 nm which disappears upon reduction by an antioxidant agent.

The DPPH solution in methanol ($6\times {10}^{-5} \mathrm{M}$) was daily prepared, and 3 ml of this solution was mixed with 100 μl of methanolic plant extract solutions. Then, the samples were incubated in a water bath for 20 min at 37 °C, and the decrease in absorbance at 515 nm was measured (A_E_). In addition, the blank sample was daily prepared in the DPPH solution containing 100 μl of methanol and its absorbance was determined (A_B_). This experiment was repeated three times. The following formula was used to quantify the radical scavenging activity.

1 $$\% \,{\text{Inhibition }} = \, \left( {\left[ {\left( {{\text{A}}_{{\text{B}}} - {\text{A}}_{{\text{E}}} } \right)/{\text{A}}_{{\text{B}}} } \right] \, \times { 1}00} \right),$$

where *A*_B_ is the absorbance of the blank sample, and *A*_E_ refers to the absorbance of the plant extract.

### Color evaluation

Color is known as a key parameter in the assessment of food products and their stability^46^. A color shift was observed during the drying due to the biochemical reactions. The reaction rates are greatly influenced by the process conditions and the drying methods^47^. The surface color values of both dried and fresh *Ganoderma* slices were characterized by a Chroma Meter Colorimeter established by Konica Minolta of Japan at room conditions. The colorimeter was calibrated to a regular black and whiteboard before the measurements. The color values represented by *L*∗ range between 0 (refers to blackness) and 100 (refers to whiteness). Moreover, there are two other color parameters including a and b which range between − a (greenness) and + a (redness) and also − b (blueness) and + b (yellowness), respectively^48^. Moreover, the Chroma (C), the total color difference (∆*E*), and Hue angle values can be determined by the following equations^49^.

2 $${\text{DE}}^{{2}} = \, ({\text{L}}_{0}^{*} - {\text{L}}^{*} )^{{2}} + \, \left( {{\text{a}}_{0}^{*} - {\text{ a}}^{*} } \right)^{{2}} + \, \left( {{\text{ b}}_{0}^{*} - {\text{ b}}^{*} } \right)^{{2}} ,$$

3 $$\mathrm{roma }=\sqrt{{a}^{*2}+{b}^{*2}},$$

4 $${\text{Hue }} = { }tan^{ - 1} { }\left( {\frac{b*}{{a*}}} \right)\quad {\text{when}}\,{\text{a }} > { }0\,{\text{and}}\,{\text{b }} > { }0,$$

where L_0_^*^, a_0_^*^, and b_0_^*^ stand for the fresh samples.

It should be mentioned that the Hue angle parameter represents the color tone (i.e., red–purple: 0°, bluish-green: 180°, yellow: 90°, and blue: 270°). Moreover, the Chroma parameter (C) uses as a standard for the color’s saturation or purity.

### Calcium determination

The samples were prepared (Washed, dried, and milled) according to the previously published literature^50^. Firstly, samples were washed with water, and then they have been washed again with 0.1 mol HCl solution and distilled water. The plant sample was dried in an oven for 48 h at 70 °C and then it was milled. The milled sample was passed through a 0.5 mm sieve and its amount of nutrient absorption such as N, P, K, Ca, and Mg was measured. The obtained extract from the freeze-drying was used to determine the amount of calcium and magnesium. In this regard, 1 ml of extracts were diluted with distilled water at a 1:9 ratio. In addition, 0.25 ml of diluted extract was transferred to a tube with a micropipette and then 4.75 ml of La(NO_3_)_3_ solution containing 1 mg of La was added to the tube. This prepared extract was used to determine the calcium absorption at 422.7 nm wavelength through an absorption spectrophotometer. The following formula was applied to calculate the calcium content in the dried plant samples.

5 $$Ca \, = \, \left( {a - b} \right) \times 1/500 \times V/W \times 100/D.M,$$

where a is the calcium concentration of extract in ppm, b is the calcium concentration of the control sample in ppm, *V* is the initial volume in ml,* W* is the sample weight in g, and *D.M* refers to the weight percentage of dried plant. It is worth mentioning that the calcium content of the fresh sample was measured at 0.2%.

### Evaluating specific energy consumption

During the preparation of dried samples in both dryers, the weight of the samples was measured. The following equation can be used to determine the specific energy consumption of the drying process in MJ/g water.

6 $$SEC=\frac{{E}_{t}}{{M}_{w}},$$

where E_t_ stands for the energy consumption of dehumidification and M_w_ refers to the weight of removed water from the samples during the drying process.

### Statistical analysis

Response surface methodology (RSM) from Design-Expert software version 12.0 with central composition design (CCD) was used to optimize the impacts of several factors including air temperature, distance, power infrared, and air velocity on the process for drying time, energy consumption, antioxidant extraction, calcium extraction, and total color difference from *Ganoderma lucidum*.

A CCD is the mostly applied category of response surface designed experiment. In addition, it is a factorial or fractional factorial design with center points, enlarged with a group of star points that can have great capability in curvature predictions. In this study, according to two considered input variables for the convective and infrared drying process, the CCD approach suggested 13 experiments and after testing these experimental conditions, modeling and optimization studies were carried out in the abovementioned software.

## Results and discussions

### Comparison of the drying time

The drying time of *Ganoderma lucidum* fruiting bodies was substantially dependent on the technique of drying, temperature, and power. The ranges of parameters in infrared and convective dryers have been reported in Tables 1 and 2.

**Table 1:** Designed experiments in the convective dryer by central composite.

Run	Air temperature (°C)	Air velocity (m/s)	Time (min)	SEC (MJ/g)	Calcium %	Total color difference ∆E	Antioxidant capacity %
1	43	0.6	575	0.9	0.1300	8.66318	0.630
2	50	0.5	730	1.368	0.1100	8.21125	1.110
3	40	1.0	605	1.26	0.1700	18.6567	0.571
4	43	1.4	330	1.4832	0.0500	14.4945	1.048
5	57	1.4	550	0.9396	0.0300	12.6896	0.833
6	60	1.0	343	0.7416	0.0500	19.6635	0.909
7	57	0.6	510	1.0404	0.2000	8.72231	0.780
8	50	1.0	490	1.2996	0.0300	11.7748	1.143
9	50	1.0	478	1.3428	0.0249	11.4356	1.147
10	50	1.0	486	1.2888	0.0280	11.3957	1.145
11	50	1.0	475	1.2636	0.0269	11.7905	1.139
12	50	1.0	480	1.2456	0.0289	11.9802	1.140
13	50	1.5	270	1.242	0.1500	8.83315	1.176

**Table 2:** Designed experiments in the infrared dryer by central composite.

Run	Infrared power (W)	Distance (cm)	Time (min)	Energy (KWh)	Calcium %	Total color difference ∆E	Antioxidant capacity %
1	1000	10	44	0.35172	0.76	8.1408	0.522
2	646	14	80	0.41328	0.874	25.5791	0.572
3	646	6	70	0.36	0.786	26.7314	0.356
4	1500	10	35	0.4176	0.269	23.9089	0.5
5	1000	4	25	0.198	0.348	18.8704	0.304
6	1354	14	40	0.43308	0.771	13.7219	0.546
7	500	10	125	0.49968	0.849	14.3301	0.636
8	1354	6	29	0.31392	0.239	22.4311	0.379
9	1000	16	53	0.42372	0.879	28.8501	0.736
10	1000	10	50	0.35172	0.73	9.3301	0.51
11	1000	10	49	0.39168	0.7	7.80939	0.511
12	1000	10	44	0.39996	0.74	8.1111	0.52
13	1000	10	43	0.342	0.8	8.39395	0.576

It was observed that the lowest drying time is related to the infrared dryer (IP = 1000 W, L = 4 cm), and the highest is related to the convective dryer (T = 50 °C, V = 0.5 m/s). The minimum drying time of the samples in convective and infrared dryers was 270 and 25 min, respectively. It means that the infrared dryer reduced the drying time by up to 90%. Heat is released from the inside of the sample by the absorption of the infrared radiation in the infrared dryer and water is transported from the inside of the sample to its surface, leading to rapid drying. These observations have been confirmed with literature^51^ for drying properties of mushroom slices and the more the power and the less the sample distances, the less the drying time in the infrared dryer. Moreover, this research indicated that the more the air velocity and temperature the less the drying time in a convective dryer. Motevali et al.^52^ figured out that increasing the air flow rate reduces vapor pressure, which results in the material’s ability to evaporate moisture faster.

Equations (7) and (8) have been obtained to predict the drying time of samples in convective and infrared dryers, respectively (Table 3).

**Table 3:** Experimental design of Infrared and convective drying of *Ganoderma*.

Dryer type	Input parameter	Coded level				
		− 1.4	− 1	0	1	1.4
Convective dryer	Temperature (X_1_), °C	40	43	50	57	60
	Velocity (X_2_), m/s	0.5	0.6	1	1.4	1.5
Infrared dryer	Power (X_3_), W	500	646	1000	1354	1500
	Lamp distance (X_4_), cm	4	6	10	14	16

According to the results obtained from ANOVA, the R^2^ values for convective and infrared dryers were 0.9990 and 0.9896, respectively.

7 $$Time = +481.80 -131.00\times X1 -162.63\times X2 +100.76\times X1\times X2 -5.68 \times X1 \times X1 +10.16 \times X2 \times X2 +222.77 X1 \times X1 \times X2 +185.80 X1\times X2\times X2,$$

8 $$Time = +46.00 -31.82\times X3 +9.90\times X4 +0.2500\times X3\times X4 +15.81\times X3 \times X3 -4.69 \times X4\times X4 -4.65 X3\times X3\times X4 +11.57 X3 \times X4\times X4,$$

where X_1_X_2_ and X_3_X_4_ refer to the temperature × velocity and power × distance, respectively. Moreover, the negative sign represents the incompatible effects while the positive sign point to the synergistic effects.

**Figure 2:**
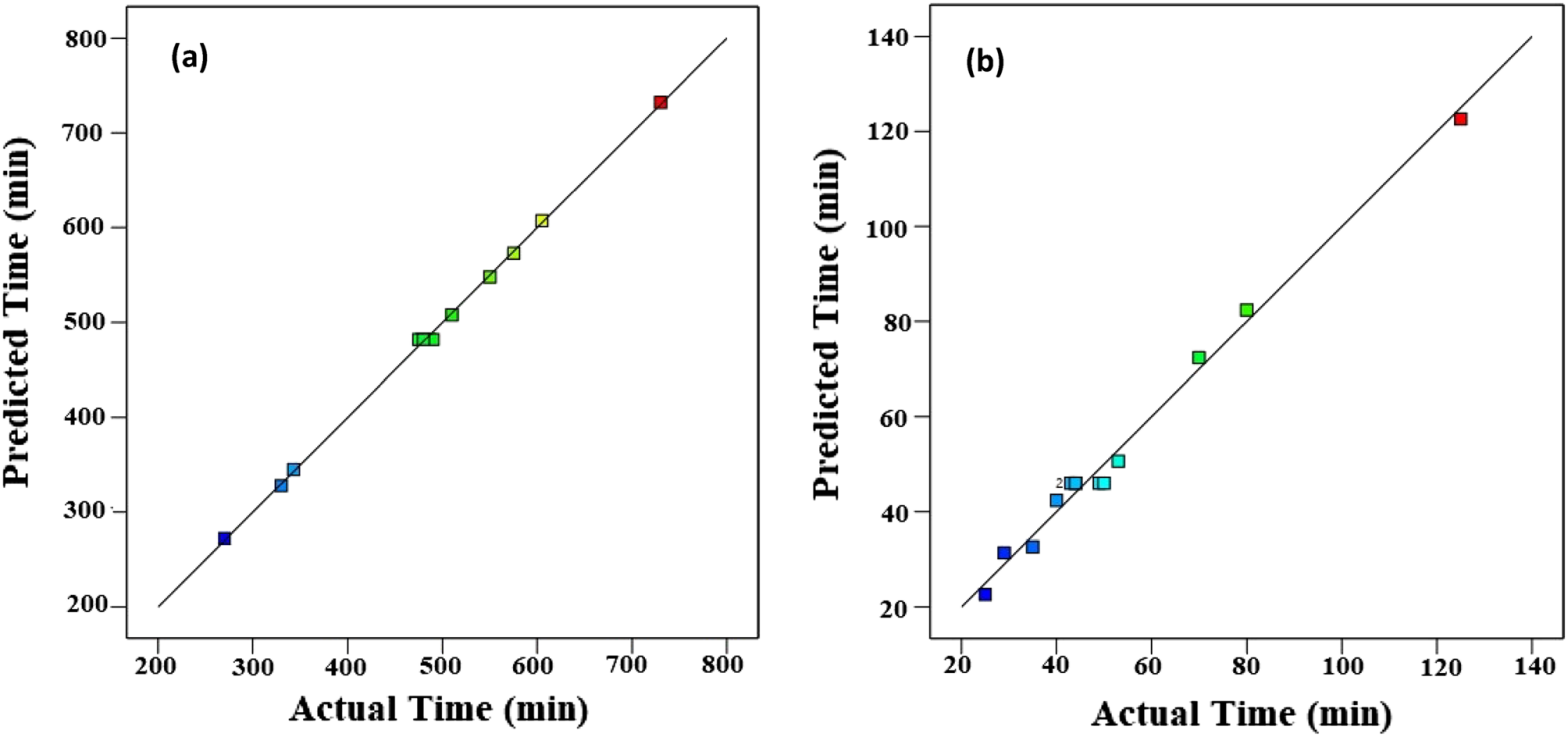
Regression plot to predict drying time for: (**a**) convective dryer and (**b**) infrared dryer.

To have a good fit by the suggested model, the significance criteria of the regression model and individual model coefficients were achieved based on the F-value or P-value with a 95% confidence level. Figure 2 indicates regression plots between predicted and actual drying time for convective and infrared dryers. As it can be seen in this figure, the actual data points illustrated by the square have a great agreement with predicted drying time values due to their closeness to the Y = X line.

**Figure 3:**
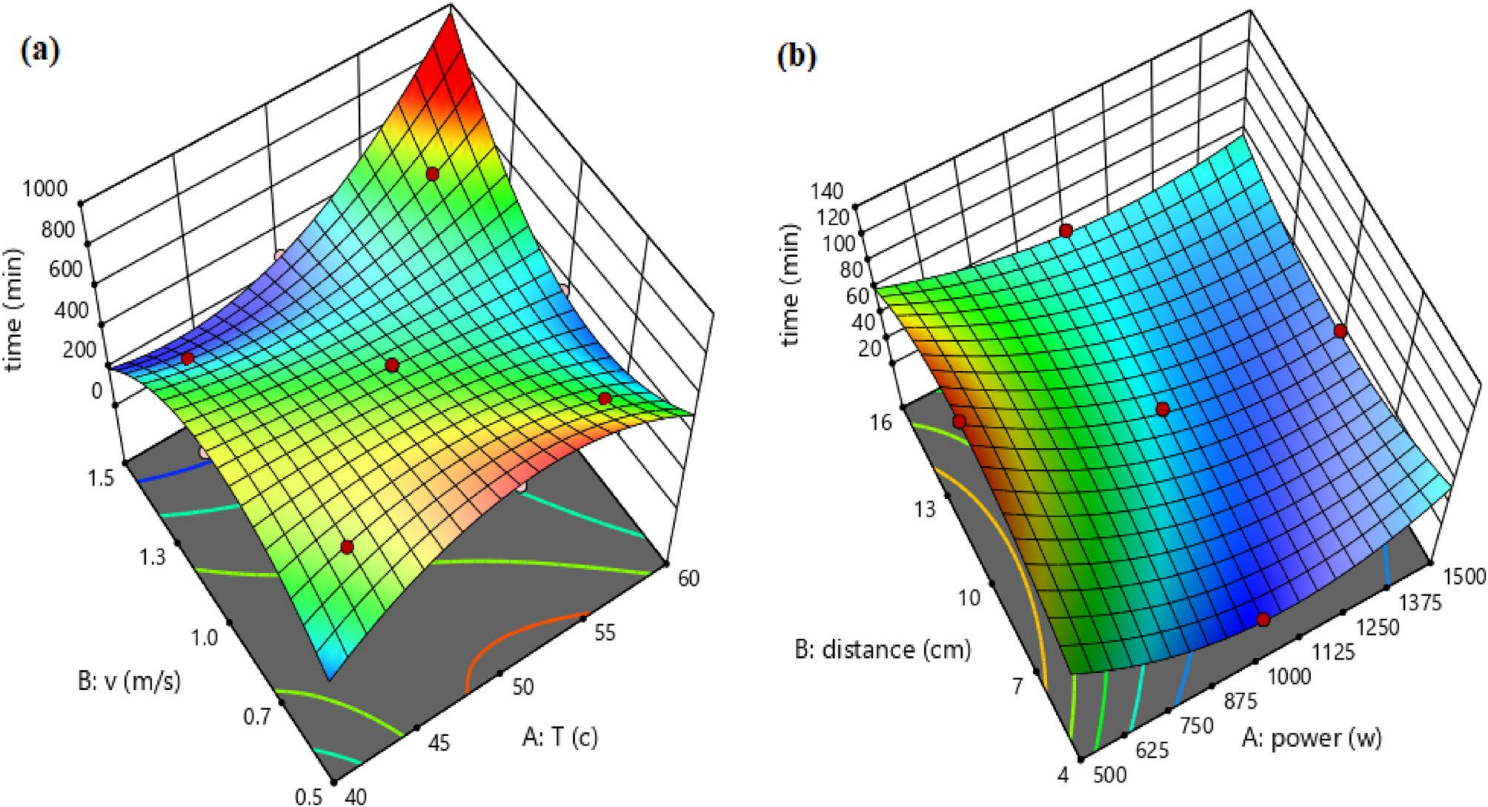
The 3-D surface graph of drying time for: (**a**) convective dryer and (**b**) infrared dryer.

In addition, the 3-D surface graph as indicated in Fig. 3 investigates the effects of temperature and velocity in the convective dryer and power and distance in the infrared dryer on the drying time.

Statistical analyses between experimental and predicted drying time for convective and infrared dryers such as R^2^, relative deviation percentage (C.V. %), F-value, and standard deviation (STD) were calculated and presented in Table 4.

**Table 4:** Statistical analyses between experimental and predicted drying time for convective and infrared dryers.

Dryer	R^2^	C.V. %	F-value	P-value	STD
Convective	0.9990	1.25	686.69	< 0.0001	6.08
Infrared	0.9896	7.90	67.67	0.0001	4.17

### Specific energy consumption (SEC)

Specific energy consumption is the energy required to remove the moisture of samples. According to Tables 1 and 2, the minimum SEC for the convective dryer was 0.7416 kWh/kg water at 60 °C and 1 m/s, while the minimum SEC for the infrared dryer obtained 0.31392 MJ/g water at 1354 W and 6 cm of lamp distance. It can be also observed that SEC values depend on drying time because as drying time be shorter, the SEC value decreases. The lowest energy required for drying mushroom slices was obtained 12.33 MJ/g water by Motevali et al.^52^ at a radiation intensity of 0.49 W/cm^2^ and air velocity of 0.5 m/s.

In addition, based on the results obtained from ANOVA, the R^2^ values for convective and infrared dryers were 0.9759 and 0.9550, respectively.

9 $$SEC = +1.29 -0.2592\times X1 -0.0445\times X2 -0.2418\times X1\times X2 -0.3183\times X1\times X1 -0.0071\times X2\times X2 +0.3303\times X1\times X1\times X2 +0.1166 \times X1\times X2\times X2,$$

10 $$SEC =+0.3647 -0.0290\times X3 +0.0798\times X4 +0.0165\times X3\times X4 +0.0444\times X3\times X3 -0.0294\times X4\times X4 -0.0367\times X3\times X3\times X4 +0.0224\times X3\times X4\times X4,$$

where X_1_X_2_ and X_3_X_4_ refer to the temperature × velocity and power × distance, respectively. Moreover, the negative sign represents the incompatible effects while the positive sign point to the synergistic effects.

**Figure 4:**
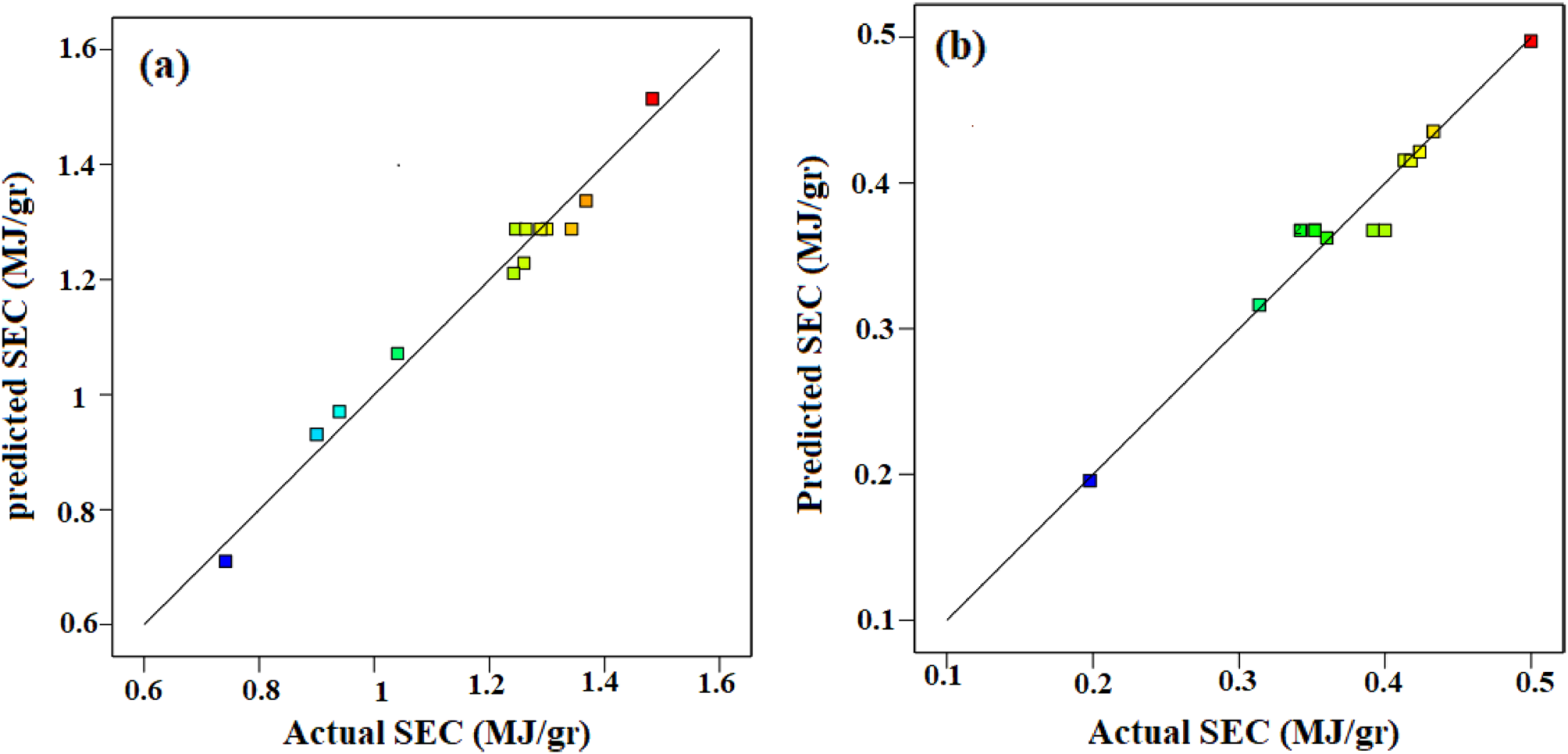
Regression plot to predict SEC for (**a**) convective dryer and (**b**) infrared dryer.

Figure 4 indicates regression plots between predicted and actual SEC for convective and infrared dryers. As it can be seen in this figure, there is a great agreement between experimental and predicted drying time values due to their closeness to the Y = X line.

**Figure 5:**
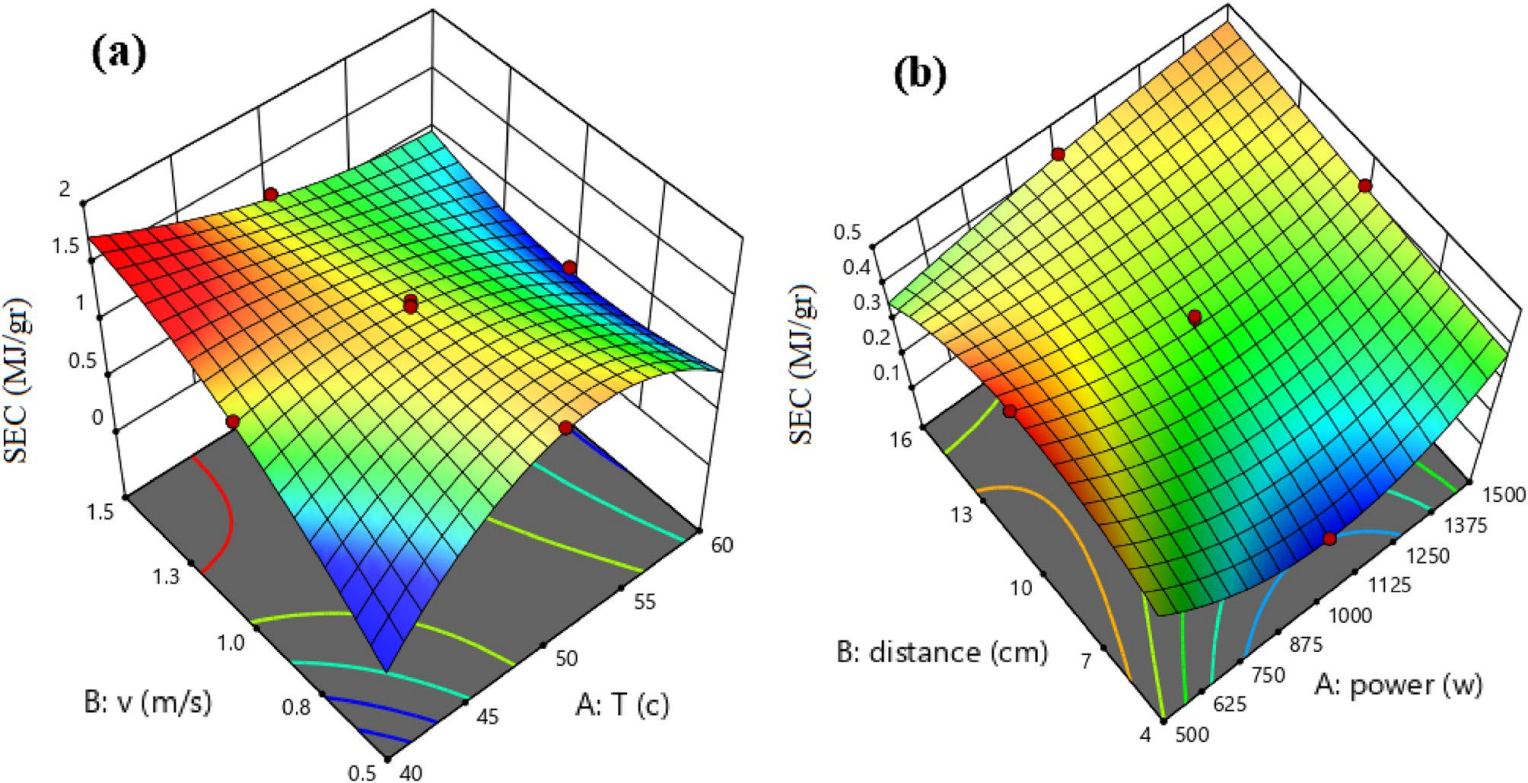
The 3-D surface graph of SEC for (**a**) convective dryer and (**b**) infrared dryer.

In addition, the 3-D surface graph as indicated in Fig. 5 to investigate the effects of temperature and velocity in convective dryer and power and distance in the infrared dryer on the SEC. In the convective dryer, the temperature has a higher impact on SEC than air velocity while an infrared dryer power has a significant impact on SEC compared to lamp distance.

Statistical analyses between experimental and predicted SEC for convective and infrared dryers such as R^2^, relative deviation percentage (C.V. %), F-value, P-value, and standard deviation (STD) were calculated and presented in Table 5.

**Table 5:** Statistical analyses between experimental and predicted SEC values for convective and infrared dryers.

Dryer	R^2^	C.V. %	F-value	P-value	STD
Convective	0.9759	4.34	28.89	0.0009	0.0515
Infrared	0.9550	6.32	15.16	0.0043	0.0238

### Influence of different drying methods on antioxidant activity

Reactive oxygen species are produced as a consequence of cellular metabolism regularly, and oxidation is considered a major contributor to several chronic degenerative illnesses^53^. *Ganoderma lucidum* exhibits substantial antioxidant activity in vitro and in vivo because of its high phenolic, triterpenoids, and polysaccharide contents^54,55^. In this study, the effects of the different drying processes on the antioxidant activity of *Ganoderma lucidum* were investigated using DPPH scavenging activity.

The capability of the suggested model was the key aim to evaluate the data analysis of the experiment. Equations (11) and (12), with R^2^ of 0.9004 and 0.9485, respectively were developed to forecast the antioxidants in infrared and convection dryers.

11 $$\mathrm{Antioxidant capacity }= +0.5278-0.0481\times \mathrm{X}3+0.1527\times \mathrm{X}4-0.0122\times \mathrm{X}3\times \mathrm{X}4-0.0001\times \mathrm{X}3\times \mathrm{X}3-0.0241\times \mathrm{X}4\times \mathrm{X}4-0.0570\times \mathrm{ X}3\times \mathrm{X}3\times \mathrm{ X}4+0.0473\times \mathrm{ X}4\times \mathrm{X}4\times \mathrm{ X}3,$$

12 $$\mathrm{Antioxidant capacity }= +1.14 +0.1690\times \mathrm{X}1 +0.0233\times \mathrm{X}2 -0.1290\times \mathrm{X}1\times \mathrm{X}2 -0.4622\times \mathrm{X}1\times \mathrm{X}1 -0.0296\times \mathrm{X}2\times \mathrm{X}2 +0.1888 \times \mathrm{X}1\times \mathrm{X}1\times \mathrm{ X}2 -0.1920\times \mathrm{ X}2\times \mathrm{X}2\times \mathrm{ X}1.$$

**Figure 6:**
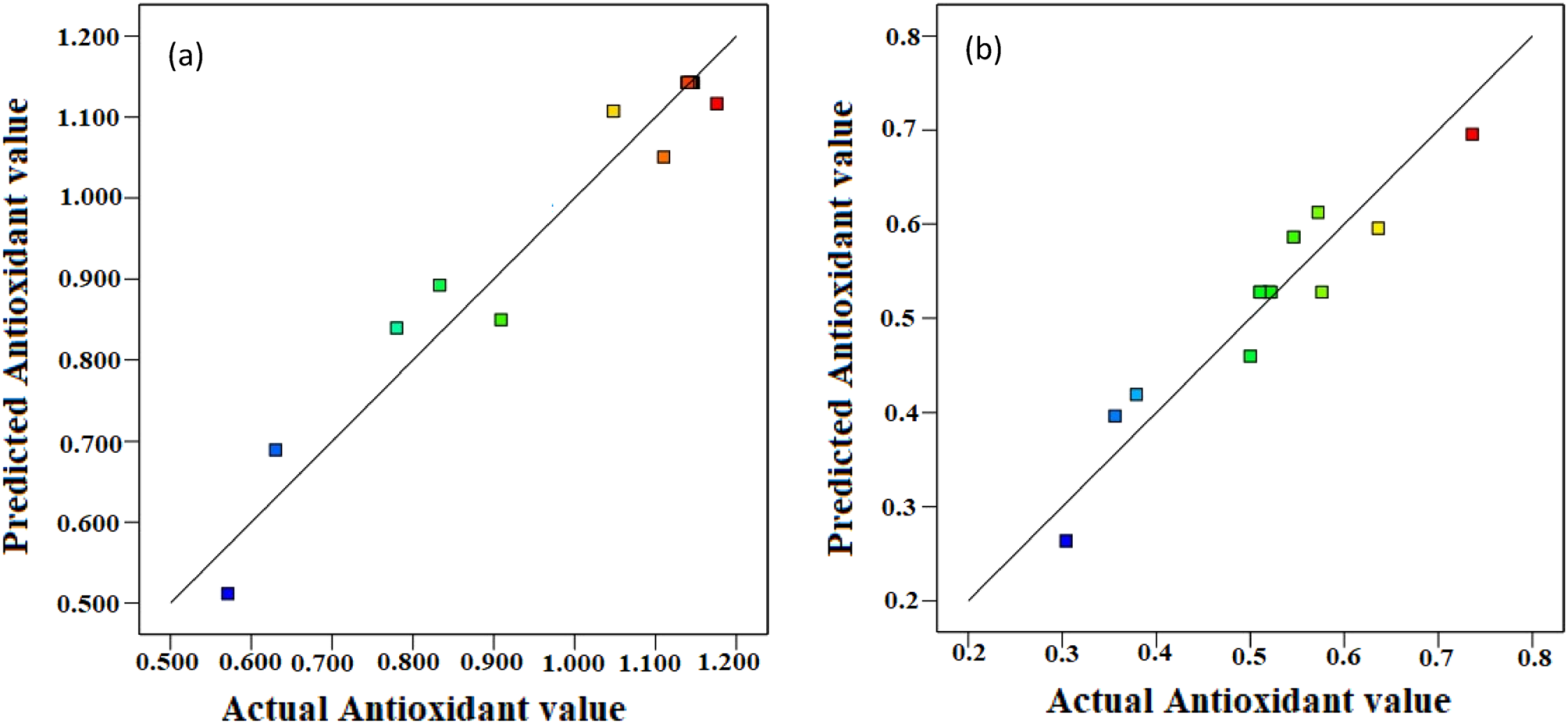
Regression plot to predict antioxidant value for (**a**) convective dryer and (**b**) infrared dryer.

Figure 6 indicates regression plots between predicted and actual antioxidant activity for convective and infrared dryers. As it can be seen in this figure, there is a great agreement between experimental and predicted antioxidant values due to their closeness to the Y = X line.

**Figure 7:**
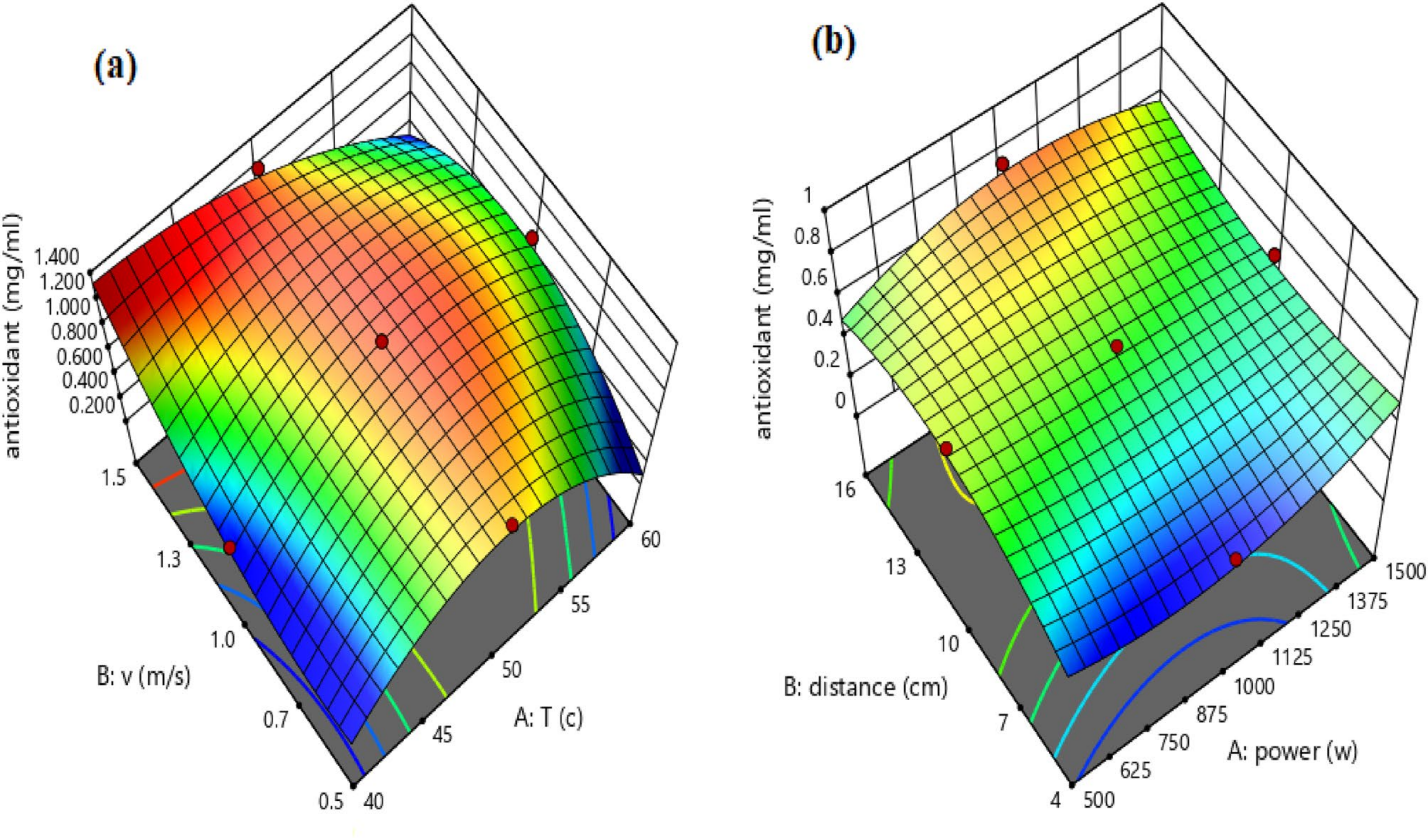
The 3-D surface graph of antioxidant value for (**a**) convective dryer and (**b**) infrared dryer.

In addition, the 3-D surface graph as indicated in Fig. 7 investigates the effects of temperature and velocity in the convective dryer and power and distance in the infrared dryer on the antioxidant values. According to Fig. 7, the ability of the free radical scavenging of DPPH in the infrared dryer was better than the convective dryer and it has been able to increase the percentage of inhibition up to 47%. An et al.^7^ confirmed that the antioxidant capacity of dried Chinese ginger by infrared dryer was higher than hot air and microwave dryers.

According to the ANOVA results, Table 3 indicated the difference in the amount of dried Ganoderma antioxidant extraction.

Statistical analyses between experimental and predicted Antioxidant activity for convective and infrared dryers such as R^2^, relative deviation percentage (C.V. %), F-value, P-value, and standard deviation (STD) were calculated and presented in Table 6.

**Table 6:** Statistical analyses between experimental and predicted Antioxidant activity for convective and infrared dryers.

Dryer	R^2^	C.V. %	F-value	P-value	STD
Convective	0.9485	7.65	13.16	0.0059	0.0725
Infrared	0.9004	11.05	6.40	0.0288	0.0567

### Color test

Color is one of the most important quality factors that impact consumer choice and establishes the final product's quality. Color changes due to drying in vegetables and fruits can be caused by non-enzymatic browning in addition to pigment loss^56^. The results indicated that at the midpoints of the range defined for power and distance, the surface color changes of the samples during drying are low. High power causes a lot of color change. As the power increases increased and the distance decreases, significant changes were observed in the color surface of the infrared dryer. In the convective dryer at constant temperatures, there were significant changes in color at high speeds and the sample was dark. *Ganoderma* samples are more sensitive to higher temperature, as seen by the increase browning of the samples that occurred when the drying temperature was raised. In the case of hot air drying, a similar phenomenon was approved by Kotwaliwale et al.^57^ and Argyropoulos et al.^58^ during drying of oyster mushroom and Boletus edulis mushrooms, respectively.

It can also be observed that at low temperatures and speeds, the color changes were low and uniform. Based on ANOVA results, the R^2^ values were 0.9973 and 0.9052 for infrared and convective dryers, respectively.

Equations (13) and (14) were developed for predicting the change in surface color of the samples in infrared and convective dryers, respectively:

13 $$\Delta \mathrm{E }= +8.36+3.39\times \mathrm{X}3+3.53\times \mathrm{X}4-1.89\times \mathrm{X}3\times \mathrm{X}4+5.54\times \mathrm{ X}3\times \mathrm{ X}3 +7.91\times \mathrm{ X}4\times \mathrm{ X}4 -5.99\times \mathrm{ X}3\times \mathrm{ X}3\times \mathrm{ X}4 -7.43 \times \mathrm{X}3 \times \mathrm{X}4\times \mathrm{ X}4,$$

14 $$\Delta \mathrm{E }= +11.68+0.5034\times \mathrm{X}1+0.2199\times \mathrm{X}2-0.6590 \times \mathrm{X}1\times \mathrm{ X}2 +6.14\times \mathrm{ X}1\times \mathrm{ X}1 -2.25\times \mathrm{ X}2\times \mathrm{ X}2 +4.46 \times \mathrm{X}1\times \mathrm{ X}1\times \mathrm{ X}2 -1.12 \times \mathrm{X}1\times \mathrm{ X}2\times \mathrm{ X}2.$$

**Figure 8:**
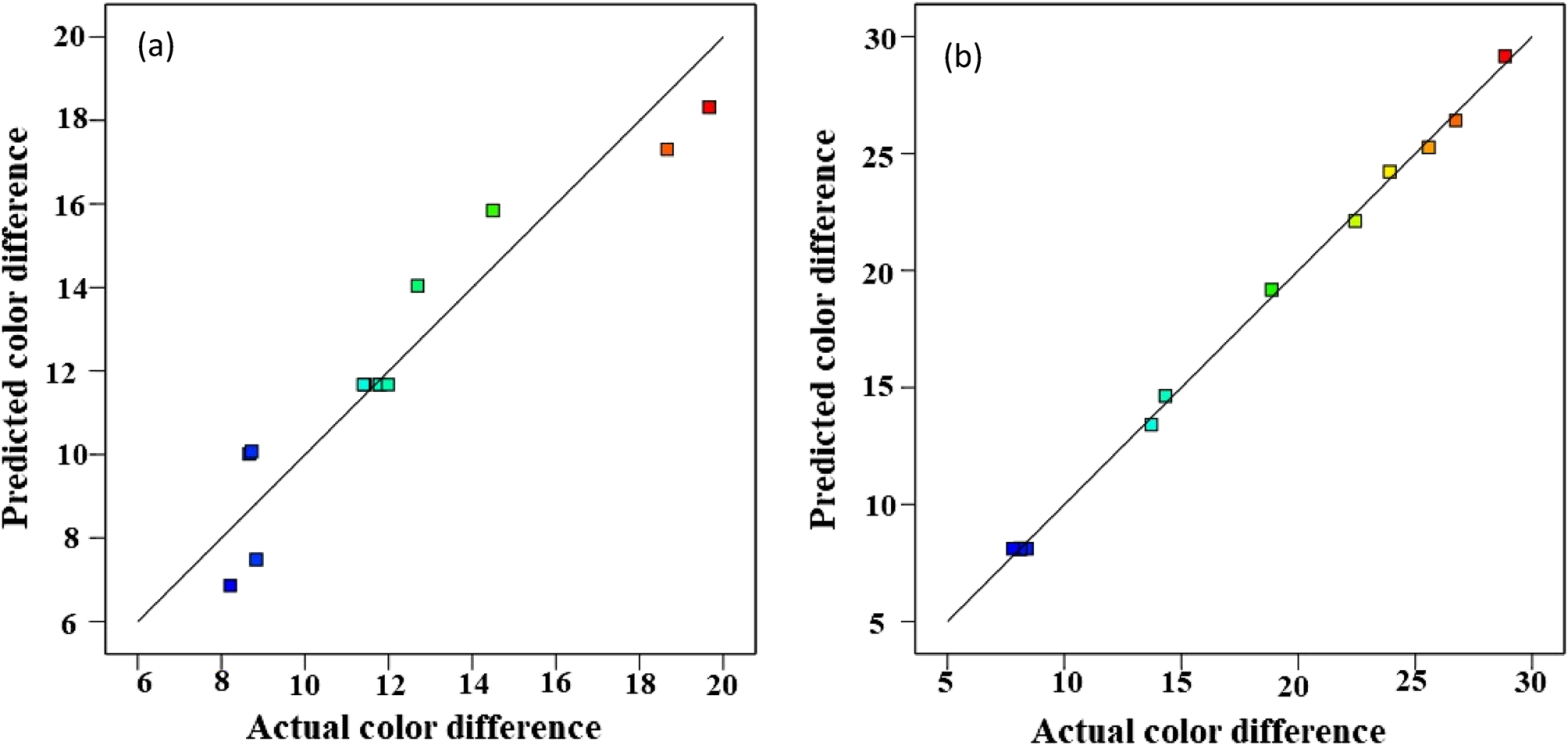
Regression plot to predict total color difference for (**a**) convective dryer and (**b**) infrared dryer.

Figure 8 indicates regression plots between predicted and actual color differences for convective and infrared dryers. As it can be seen in this figure, there is a great agreement between experimental and predicted color difference values due to their closeness to the Y = X line.

**Figure 9:**
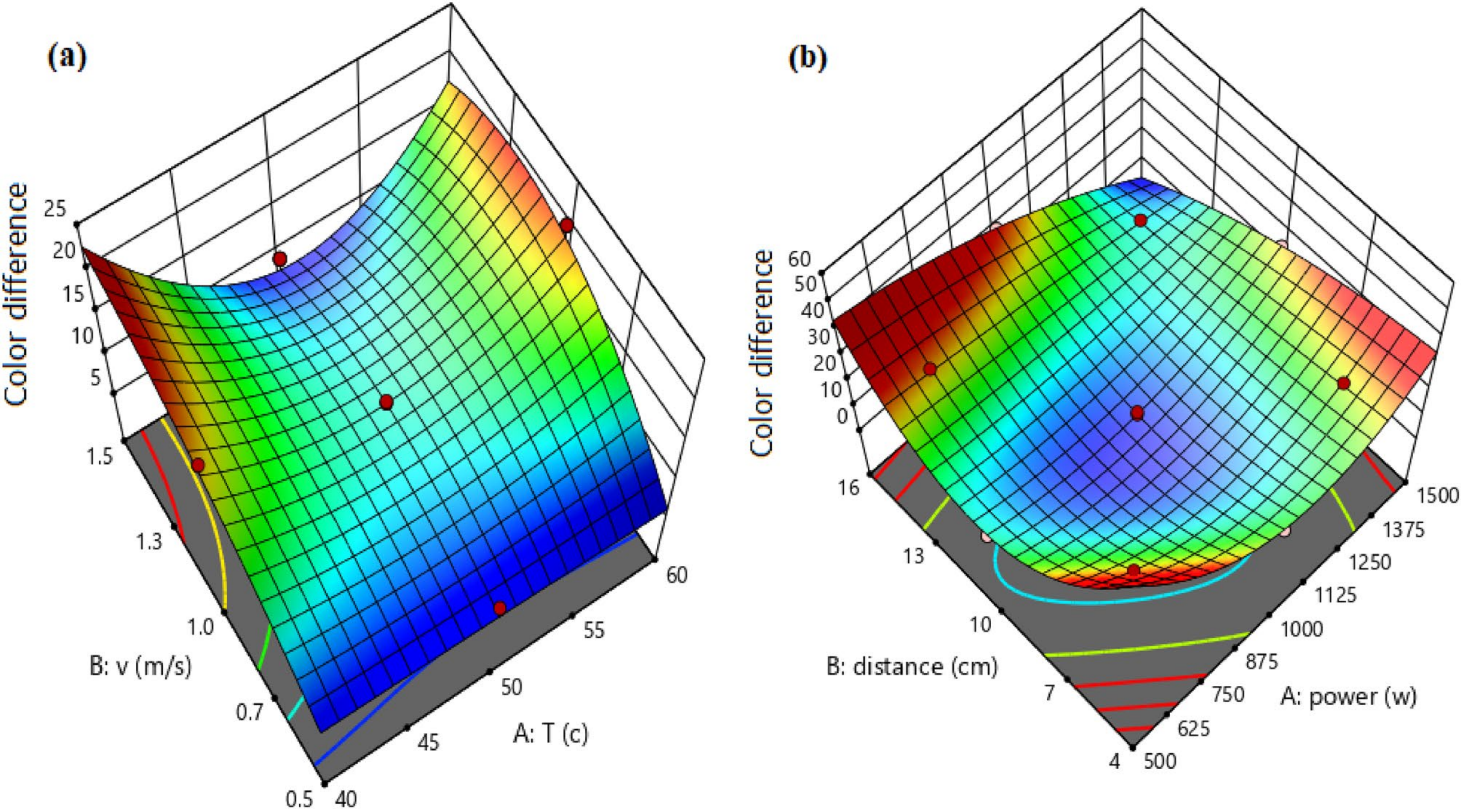
The 3-D surface graph of total color difference for (**a**) convective dryer and (**b**) infrared dryer.

In addition, the 3-D surface graph as indicated in Fig. 9 investigates the effects of temperature and velocity in the convective dryer and power and distance in the infrared dryer on the color difference values.

Statistical analyses between experimental and predicted total color differences for convective and infrared dryers such as R^2^, relative deviation percentage (C.V. %), F-value, P-value, and standard deviation (STD) were calculated and presented in Table 7.

**Table 7:** Statistical analyses between experimental and predicted total color differences for convective and infrared dryers.

Dryer	R^2^	C.V. %	F-value	P-value	STD
Convective	0.9052	14.14	6.82	0.0252	1.72
Infrared	0.9973	3.93	259.88	< 0.0001	0.6541

### Calcium test

According to the results reported in Tables 1 and 2, the preservation of calcium compounds in the infrared dryer was much better than convective. The amount of calcium in fresh Ganoderma is 0.2.

Equations (15) and (16) have been written for the estimation of the calcium content of the dried Ganoderma in convective and infrared dryers, respectively.

15 $$\mathrm{Calcium }= +0.0277 -0.0600\times \mathrm{X}1 +0.0141\times \mathrm{X}2 -0.0318 \times \mathrm{X}1\times \mathrm{ X}2 +0.0735 \times \mathrm{X}1\times \mathrm{ X}1 +0.0468 \times \mathrm{X}2\times \mathrm{ X}2 -0.1533\times \mathrm{ X}1\times \mathrm{ X}1\times \mathrm{ X}2 +0.0777\times \mathrm{ X}2\times \mathrm{ X}2\times \mathrm{ X}1,$$

16 $$\mathrm{Calcium }= +0.7460 -0.2051\times \mathrm{X}3 +0.1877\times \mathrm{X}4 +0.1110 \times \mathrm{X}3\times \mathrm{ X}4 -0.0732\times \mathrm{X}3\times \mathrm{ X}3 -0.0459\times \mathrm{ X}4\times \mathrm{ X}4 -0.0327\times \mathrm{ X}3\times \mathrm{ X}3\times \mathrm{ X}4 +0.0426\times \mathrm{ X}3\times \mathrm{ X}4\times \mathrm{ X}4.$$

**Figure 10:**
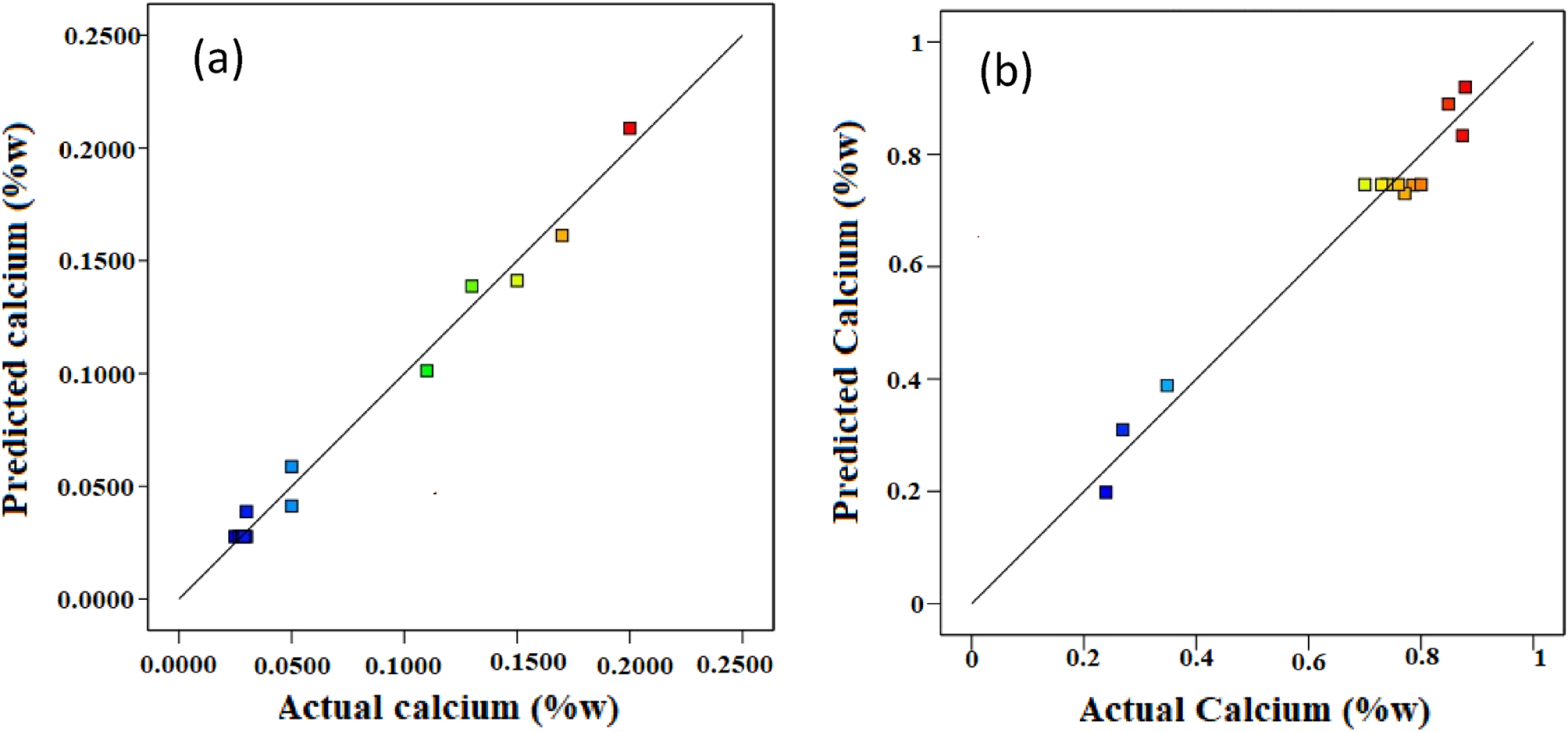
Regression plot to predict calcium for: (**a**) convective dryer and (**b**) infrared dryer.

Figure 10 indicates regression plots between predicted and actual calcium values for convective and infrared dryers. As it can be seen in this figure, there is a great agreement between experimental and predicted calcium values due to their closeness to the Y = X line.

**Figure 11:**
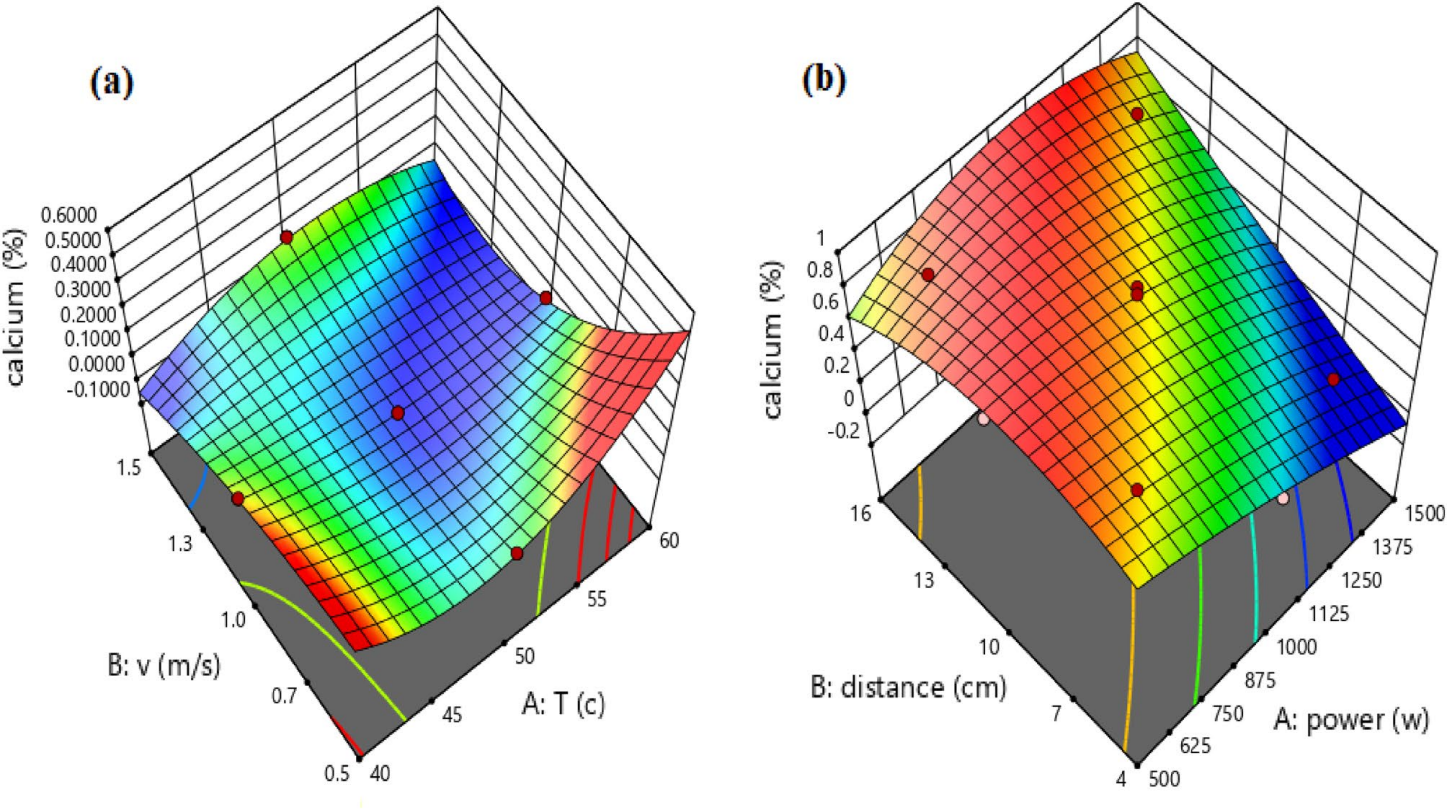
The 3-D surface graph of calcium for (**a**) convective dryer and (**b**) infrared dryer.

In addition, the 3-D surface graph as indicated in Fig. 11 investigates the effects of temperature and velocity in the convective dryer and power and distance in the infrared dryer on the calcium values.

As shown in Fig. 11, at lower powers and longer distances, the calcium contents in dried samples were higher. In addition, high power causes calcium to be lost in the infrared dryer. In addition, in a convective dryer at a constant speed, better responses were obtained at the beginning and end of the temperatures. It means that at 40 °C and 60 °C, there were higher calcium contents and also at the lowest speed, this calcium content was much higher. This finding was confirmed with Rongchai et al.^59^ article regarding determination of the calcium content in dried moringa leaves. They indicated that in the lower temperature, the calcium contents were increased.

Statistical analyses between experimental and predicted calcium content for convective and infrared dryers such as R^2^, relative deviation percentage (C.V. %), F-value, P-value, and standard deviation (STD) were calculated and presented in Table 8.

**Table 8:** Statistical analyses between experimental and predicted calcium content for convective and infrared dryers.

Dryer	R^2^	C.V. %	F-value	P-value	STD
Convective	0.9871	14.16	54.76	0.0002	0.0112
Infrared	0.9701	9.10	23.15	0.0016	0.0612

**Figure 12:**
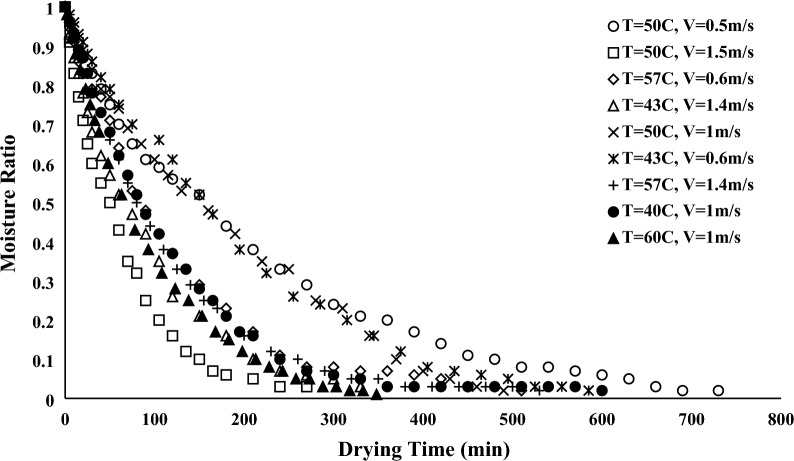
Moisture ratio versus drying time for convective dryer.

**Figure 13:**
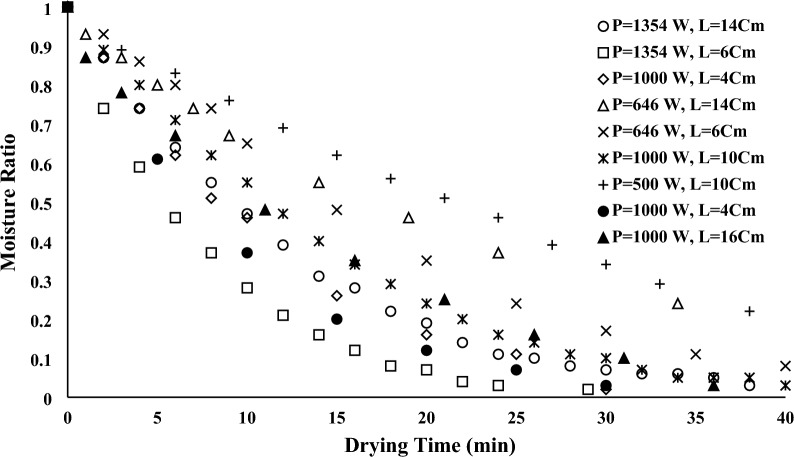
Moisture ratio versus drying time for infrared dryer.

The moisture ratio during the drying process, which indicates a reduction in the moisture of samples, was measured to investigate the kinetics of drying. In addition, Figs. 12 and 13 indicated moisture ratios versus drying times for convective and infrared dryers, respectively. As it can be seen, at constant drying time, the moisture ratio for the convective dryer is more than the infrared dryer because the infrared dryer reduces drying time, which is the advantage of the infrared dryer compared to the convective dryer. The following formula can be used to determine the moisture ratio^39^.

17 $$MR\left(\%\right)=\frac{Mt-Me}{{M}_{0}-Me}\times 100.$$

M_t_ and M_0_ refer to the time-dependent and preliminary weight of the sample, respectively and M_e_ is the weight of the sample at EMC condition.

As it can be seen in moisture curves, at a constant velocity, samples dried with higher temperatures have lower drying times for convective dryers. In addition, drying time can be increased in the infrared dryer when the lamp distance increased in constant power.

### Optimization procedure

**Figure 14:**
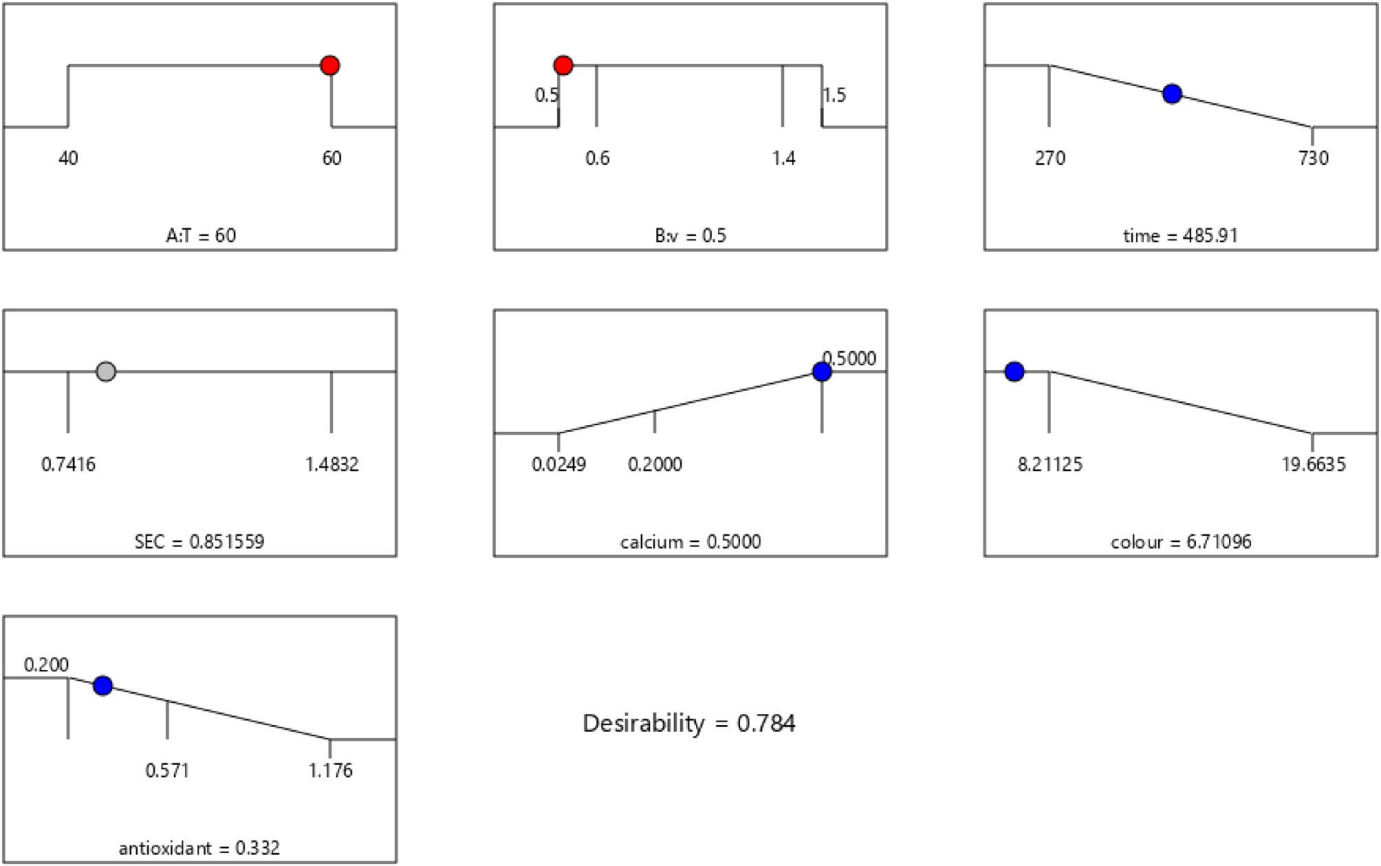
Optimization point for convective dryer.

**Figure 15:**
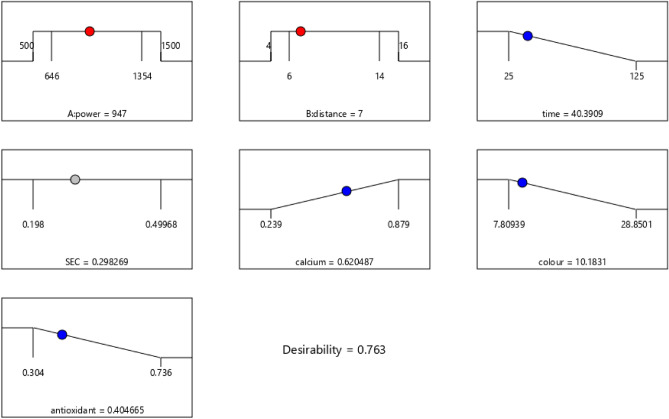
Optimization point for infrared dryer.

Maximum antioxidant and calcium contents and minimum color difference and drying time were supposed as critical criteria for optimizing the drying processes. The solutions for the optimum coverage of the criteria were given using the desirability function approach. The ideal values of all three variables, with the highest desirability coefficient, were 0.5 m/s and 60 °C in the convective dryer and 947 W, and 7 cm in the infrared dryer. At this point, the antioxidant, calcium content, time, and color difference were expected to be 0.332*%*, 0.5%, 485.91 min, and 6.71, respectively in the convective dryer (see Fig. 14) and 0.404%, 0.62%, 40.39 min, and 10.183, respectively in the infrared dryer (see Fig. 15).

**Figure 16:**
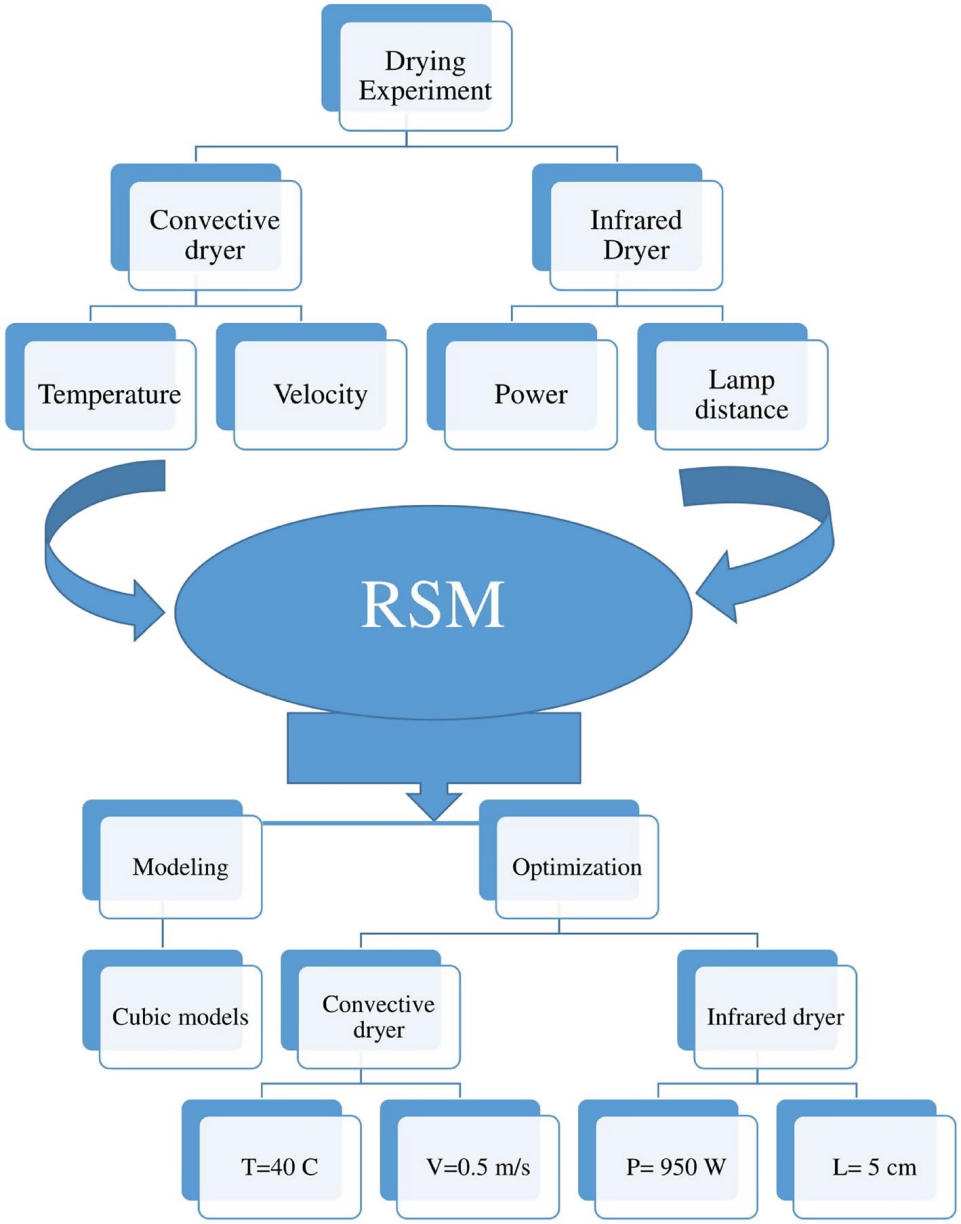
Flowchart of the drying process in this study.

As a brief configuration of the current study, Fig. 16 indicates a flowchart of the drying process by convective and infrared dryers and also indicates the progress of modeling and optimization method carried out by the RSM approach.

## Conclusions

The present study aimed to investigate different characteristics of *Ganoderma lucidum* such as calcium, total color difference, and antioxidant values dried by the convective dryer and infrared dryer. Experimental investigation of these properties accompanied by modeling the drying process is the novelty of current contribution. The measurable parameters in the convective dryer were temperature and air velocity while lamp distance and power were influential variables in the infrared dryer. The results indicated that the infrared dryer has a better effect on drying time, energy consumption, amount of calcium, and antioxidants. The calcium content of the dried sample in the infrared dryer increased from 0.2 to 0.62% compared to the fresh sample, while this property increased from 0.2 to 0.5% in convective dryer. In the infrared dryer, the amount of antioxidants extracted was significantly higher than in the convective dryer. The highest amount of calcium was obtained at low power and distance and the highest amount of antioxidants was obtained at high power and short distance. Moreover, increasing the power and reducing the distance had remarkable effects in reducing the drying time and energy consumption. The surface color changes of the samples were less in the convective dryer. In addition, ANOVA was used to develop formulas for predicting antioxidant, calcium content, drying time, and color difference values. The optimal conditions were obtained at the distance of 7 cm and power of 947 W in the infrared dryer while this condition was obtained at an air velocity of 0.5 m/s and air temperature of 60 °C in the convective dryer. Consequently, this research presents an economical process to obtain high value added medical products and it can help researchers who deal with herbal medicines how can dry materials in the best conditions.

## Data Availability

The authors declare that the data supporting the findings of this study are available within the paper. If any raw data files be needed in another format they are available from the corresponding author upon reasonable request.
